# Development and validation of a Malawian version of the primary care assessment tool

**DOI:** 10.1186/s12875-018-0763-0

**Published:** 2018-05-16

**Authors:** Luckson Dullie, Eivind Meland, Øystein Hetlevik, Thomas Mildestvedt, Sturla Gjesdal

**Affiliations:** 1 Department of Global Public Health and Primary Care, University of Bergen, Bergen, Norway; 2 Partners In Health, Neno, Malawi; 3 University of Malawi College of Medicine, Blantyre, Malawi

**Keywords:** Primary care, Primary care assessment tool, Patient centeredness, Patient experience, Primary care quality measurement

## Abstract

**Background:**

Malawi does not have validated tools for assessing primary care performance from patients’ experience. The aim of this study was to develop a Malawian version of Primary Care Assessment Tool (PCAT-Mw) and to evaluate its reliability and validity in the assessment of the core primary care dimensions from adult patients’ perspective in Malawi.

**Methods:**

A team of experts assessed the South African version of the primary care assessment tool (ZA-PCAT) for face and content validity. The adapted questionnaire underwent forward and backward translation and a pilot study. The tool was then used in an interviewer administered cross-sectional survey in Neno district, Malawi, to test validity and reliability. Exploratory factor analysis was performed on a random half of the sample to evaluate internal consistency, reliability and construct validity of items and scales. The identified constructs were then tested with confirmatory factor analysis. Likert scale assumption testing and descriptive statistics were done on the final factor structure. The PCAT-Mw was further tested for intra-rater and inter-rater reliability.

**Results:**

From the responses of 631 patients, a 29-item PCAT-Mw was constructed comprising seven multi-item scales, representing five primary care dimensions (first contact, continuity, comprehensiveness, coordination and community orientation). All the seven scales achieved good internal consistency, item-total correlations and construct validity. Cronbach’s alpha coefficient ranged from 0.66 to 0.91. A satisfactory goodness of fit model was achieved (GFI = 0.90, CFI = 0.91, RMSEA = 0.05, PCLOSE = 0.65). The full range of possible scores was observed for all scales. Scaling assumptions tests were achieved for all except the two comprehensiveness scales. Intra-class correlation coefficient (ICC) was 0.90 (*n* = 44, 95% CI 0.81–0.94, *p* < 0.001) for intra-rater reliability and 0.84 (*n* = 42, 95% CI 0.71–0.96, *p* < 0.001) for inter-rater reliability.

**Conclusions:**

Comprehensive metric analyses supported the reliability and validity of PCAT-Mw in assessing the core concepts of primary care from adult patients’ experience. This tool could be used for health service research in primary care in Malawi.

**Electronic supplementary material:**

The online version of this article (10.1186/s12875-018-0763-0) contains supplementary material, which is available to authorized users.

## Background

Evidence from both developed and developing countries indicates that well established primary care is the backbone of effective, efficient and equitable health care delivery systems [1–7]. Investing more in primary health care interventions is likely to accelerate progress towards achieving the sustainable development goal of universal health coverage [8]. A growing focus is also emerging to investigate primary care performance and organization in different settings using data from patients’ assessment of service delivery [9–12].

Malawi is a signatory to global declarations on primary health care and has a health sector strategic plan “that is inspired by the primary health care approach” [13]. Malawi’s health system is faced with the most severe shortage of healthcare personnel in sub-Saharan Africa with only two (2) physicians and 34 nurse/midwives per 100,000 inhabitants [14]. Mid-level health care workers such as clinical officers and medical assistants form the bulk of the work force as providers of primary care [15]. Most health indicators, while slowly improving, remain poor. Access, equity and financial risk protection are still major challenges [14–16].

There are three levels of health care in Malawi. Primary care consists of dispensaries and health centers which target a coverage radius of 8 km. Secondary level care is provided in district hospitals while tertiary care is delivered in three regional and two mental hospitals. There is an essential health package of services since 2004 that is offered in all public facilities as well as those belonging to the faith based organizations. Patients enter the system at first level and are referred higher up depending on the need [13].

To augment this primary health care structure, Malawi’s sole medical school has since 2015 started a specialist family medicine training program to train family physicians who will lead district health systems towards primary health care implementation. This approach is already showing evidence of positive impact on health systems elsewhere in sub-Saharan Africa [17, 18]. Earlier similar findings have come from developed and mid-level emerging countries like China and Brazil [19].

The Ministry of Health in Malawi has established a memorandum of understanding with the non-governmental organization Partners In Health to use the rural district of Neno in the South-west part of the country as a model of primary care delivery. As a result, novel models of primary care interventions are being implemented in the district to reflect program integration of programmatic interventions, [20] community orientation [21] and financial risk protection [22].

As an integral part of these primary care reforms, there is need for assessment of primary care performance in order to describe, compare and follow-up services from patients’ perspectives. Several instruments have been developed in order to make this assessment structured and standardized way in different settings [23–27]. Some instruments assess many aspects of primary care services (or key dimensions) whereas others only target specific dimensions, like accessibility or continuity of care [28].

Within primary health care research, the US Primary Care Assessment Tool (PCAT) has been widely adapted and used in patient surveys in many countries including South Africa [29–34]. Based on the 1994 American Institute of Medicine’s definition of primary care [35], the PCAT aims at a global assessment of primary care organizations and their achievements around the core dimensions of accessibility, comprehensiveness, coordination and continuity, and accountability. In addition, it also assesses derivative dimensions of family orientation, community orientation, and cultural competence.

The aim of this study was therefore to develop a reliable and valid instrument that could be used to assess primary care performance from adult patients’ perspective of the Malawian health system in order to facilitate future evaluation of heath care services and to compare performance and development over time. The Specific objectives were to adapt the South African PCAT (ZA-PCAT) to the Malawian health system and culture, and to analyze its feasibility, reliability and validity.

## Methods

### Instrument

The ZA-PCAT questionnaire is similar to the original American PCAT. Through 114 items, it measures eight domains of primary care: first contact (access and utilization), on-going care, coordination (patient care and information systems), comprehensiveness (services available and services provided), family orientation, community orientation, cultural competence and primary care team. Each item is answered on a 4-point Likert-type scale (1 = definitely not; 2 = probably not; 3 = probably; 4 = definitely) with an additional possibility to respond “not sure”. The questionnaire includes 26 additional questions to determine the user’s primary care facility/person and socio-demographic data. The ZA-PCAT was chosen for the study because of proximity and similarity of health systems to the study setting. Adapted versions of the PCAT have been used to measure primary care organization and performance, and to assess performance of primary care in different settings [9–11].

### Face and content validity

**Fig. 1:**
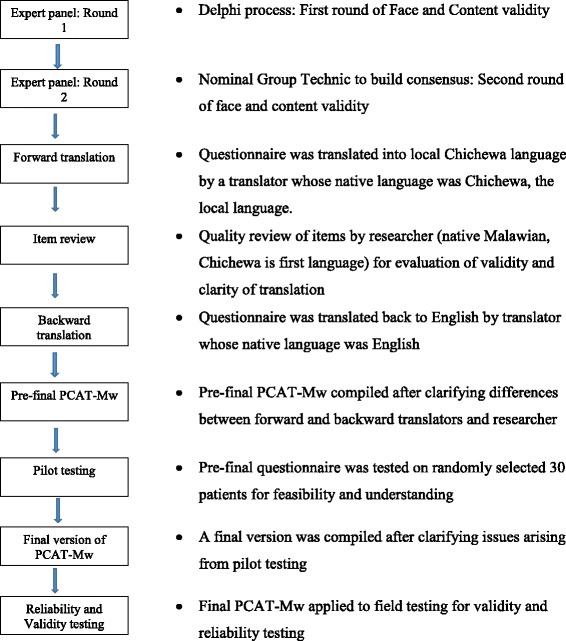
Process of cross cultural validation from ZA-PCAT to PCAT-Mw before metric analysis

The cross cultural validation from ZA-PCAT to PCAT-Mw is illustrated in Fig. 1.

Face and content validity of the questionnaire were assessed through a modified Delphi [36] and nominal group technique process [37] using a panel of 9 experts that included 2 primary care providers, 2 primary care managers, 2 primary care policy makers, 2 Family Medicine academics and 1 patient representative. The ZA-PCAT was sent to the 9 experts by e-mail. To assess content validity, each expert was asked to rate each dimension and item for relevance to the Malawi health system on Likert scale: 5 – highly relevant, 4 – relevant, 3 – not decided, 2 – not relevant, 1- highly irrelevant. Additionally, experts were asked if items were appropriately phrased and if there were additional dimensions or items to be added. Criteria for retention was at least 7 experts scoring 4 and above while exclusion was when at least 7 experts scored 2 or 1. Dimension and items with any other score results, additional dimensions and items proposed and suggested rephrasing of items were brought for the nominal group technique session using the same group of experts convened by three of the investigators. During this session, suggested new phrasing and items were discussed and experts were asked to reassess those items that had not achieved adequate consensus during the first round. Criteria for inclusion or exclusion were as described above.

For face validity, we used the definition “the degree to which a measurement instrument looks as though it is an adequate reflection of the construct to be measured” [38] and thus asked each expert to indicate whether or not the questionnaire was generally adequate to be used in the Malawian context. Results were collated to form the questionnaire that was to be translated.

### Translation and cultural adaptation

Forward translation was done by a translator whose native language was Chichewa, the most widely spoken national language (used by about 65% of the population) which was to be used in the study. A review was done by the principal investigator, a native Malawian with Chichewa as first language for clarity of the translation. A backward translation was then done by a translator whose native language was English. Any differences were sorted out through a reconciliation discussion between the translators and the principal investigator.

### Feasibility and understanding of the questionnaire- pilot testing

Six interviewers with prior experience in patient interviews were trained in the PCAT interviews. The interviewers administered the questionnaire to 30 randomly selected patients at Neno district hospital out-patient clinic. In addition to responding to the items, patients were also asked for comprehensibility of the questions, the overall relevance of the items to the Malawi setting and for suggestions for any changes to the wording. The pilot study also assessed how long the questionnaire took to complete and the feasibility of carrying interviews in the out-patient clinic. From this phase a version was obtained which was used for the survey.

### Data collection, setting and study population

A cross sectional study was carried out in August –September, 2016 in Neno, a rural district in South-West of Malawi with a population of 150,000 people, two hospitals and 11 health centers. Out-patient clinics in the two hospitals and 8 health centers were selected based on high patient volumes. Study participants were at least 18 years of age, must have been using the facility for at least six months and must have visited the facility for at least 3 times. Patients that were acutely ill, frail looking or with severe mental health disorders were excluded in order to allow for the immediate medical attention that they needed. Sample size was calculated based on similar studies using at least 5:1 subject to item ratio [30–34]. Sample size of 600 was targeted. From this it was calculated that each interviewer needed to administer seven questionnaires per day. The sampling frame was the 40–50 patients waiting to be seen on each working day. These patients were asked for permission to participate in the interview with a full explanation of the research purpose and were told that the survey would not influence their consultation. The sampling interval was calculated by dividing the number of available waiting patients by seven. The random starting point was identified using a smart phone random number generator.

### Statistical analysis

Data were entered into and analyzed using the IBM SPSS Statistics 24.0.0 (2016) package. For consistency with methods used in PCAT studies in other countries, a mid-scale value of 2.5 was assigned to “not sure” answers while the mean item score was used for missing data [26, 29–31].

First, each item responses were inspected for floor or ceiling effect and a correlation analysis was run to ensure sufficient correlation between the items.

Secondly, the data file was split randomly into 50% subsets to allow for exploratory factor analysis with sample 1 and confirmatory factor analysis with sample 2.

Prior to exploratory factor analysis of sample 1, the overall Kaiser-Meyer-Olkin (KMO) statistic and Bartlett’s test for sphericity were calculated to evaluate whether the sample was large enough to perform a satisfactory factor analysis. The KMO statistic is a measure of the shared variance in the items to justify factor analysis. On a range of 0 to 1, the desirable result is closer to 1 and the minimum recommended value is 0.6 [39]. Bartlett’s test is a chi squared test whose null hypothesis states that there are no relationships between the items. A significant test confirms that linear combinations exist between the items and that the matrix is suitable for factor analysis [40]. Factor extraction was done through principal axis factoring and varimax rotation. Principal axis factoring was chosen because it allows for the exploration of underlying constructs, which cannot be measured directly, through items thought to be reflective measures of the construct especially where there are few items per component and low component loadings [41]. Theoretically, oblique rotation should be used in the case where factors were assumed to possess underlying correlations [41]. However, the varimax rotation rendered the matrix more reproducible and easier to interpret.

Determining scale structure and item reduction was based on multiple steps. First the scree plot, which is a graphical representation of the factors and their corresponding eigenvalues, was used. Factors above the bend or elbow cut-off point were retained. Additionally, items were retained when they attained factor loadings of at least 0.32, without cross loadings of the same significance and shared the same underlying meaning of construct and had inter-item correlation between 0.2 and 0.5.

Next, internal consistency was assessed by Cronbach’s alpha and item-total correlation. For a scale to be considered sufficiently reliable, minimum Chronbach’s alpha value of 0.5 is accepted as adequate. Within the scale, all the retained items were to exceed the minimum acceptable item-total correlation of 0.30 [39].

Likert scaling assumptions were tested by assessment of equal item convergence through the range of item-total correlation; domain score reliability through Cronbach’s alpha; item-convergent validity through item-scale correlations (minimum 0.3); and item-discriminant validity using scaling success rate (correlation of each item with other items within the same scale being greater than with items from different scales).

Construct validity was analyzed throughout the measures of convergent validity and discriminant validity explained above. Further construct cross-validation was done through confirmatory factor analysis (CFA) using IBM Amos Graphics package 24.0.0 (2016) on sample 2 which was subjected to structural equation modeling. Maximum likelihood estimation was chosen with output of squared multiple correlations, maximization history, standardized estimates and index modification. The model’s overall goodness of fit was assessed using a combination of indices: chi squared test, goodness of fit index (GFI), the root mean square error of approximation (RMSEA), and an incremental fit index, the comparative fit index (CFI). Some authors advocate for an insignificant chi squared test to show model fitness [42]. This is known to be unlikely possible especially when a large sample size is used [43]. The GFI was created as an alternative to the Chi squared test and calculates the proportion of variance that is accounted for by the estimated population covariance. The statistic ranges from 0 to 1 and a minimum cut off of 0.9 is recommended [44]. RMSEA estimates how well the model would fit the sample if optimal parameters were available and uses the chi squared statistics taking degrees of freedom into account. Most authors will accept values below 0.08 but recommend those under 0.06 to indicate a sufficient fit between the specified model and the data [45]. The CFI evaluates the difference between an independent model and a specified model without being affected by the sample size and values > 0.9 are acceptable [45].

Lastly, descriptive statistics were performed for the revised PCAT domains, including the mean, standard deviation, range, skewness and kurtosis. The results of the study were planned for both local and international dissemination through meetings with local authorities, scientific conference presentations and publication in an appropriate journal.

### Further reliability tests

A subset of patients had second interviews after 4 weeks to assess consistency of the item scores through intra-rater and inter-rater reliability analysis. To do this 2 of the 10 facilities where data was collected were selected randomly. One was assigned for test –retest intra-rater reliability and patients from this facility were asked to return for a second interview by the same interviewer after 4 weeks. At the inter-rater facility, patients were asked to return after 4 weeks and were interviewed by a different interviewer from the one who did the first. Intra-class correlation coefficient (ICC) was calculated for the sum scores of the domain means of the responses of the participants with the two rounds of interviews to measure intra-rater and inter-rater reliability.

## Results

### Face and content validity

The ZA PCAT was rated to be generally relevant to the Malawi health system. Table 1 compares the item and domain structures of the ZA PCAT and the initial version of the PCAT-Mw. The general structure and content was largely similar. The modified Delphi and nominal group technique process eliminated the domain “primary care team” and modified “coordination – Health information” because patients in Malawi use patient held health passports for their medical records. There was also substitution of services available and provided to fit context in Malawi.

**Table 1:** Comparison of number of items and structure of ZA-PCAT and PCAT-Mw

Parts of the Questionnaire	ZA-PCAT	PCAT-Mw before metric analysis	Final PCAT-Mw
Core domains			
B - First contact: utilization	3	3	
C - First contact: access	19	18	3
D - Continuity of care	15	16 (plus 2 open question)	8
E - Coordination	10	9 (plus 7 open questions)	3
F - Coordination – Health information	3	4	
G - Comprehensiveness			
Services available	28	28	6
H - Comprehensiveness			
Services provided	15	14	6
Ancillary domains:			
I - Family orientation	3	3	
J - Community orientation	6	6	3
K - Cultural competence	5	5	
P - Primary care team	7		
About PC provider information	8	8	8
Socio-demographic data	18	18	18
Core domains (B-H)	93	92	26
All domains (B-P)	114	106	29
Total:	140	132 (plus 9 open questions)	47

### Pilot study

During the pilot study, it was found that the questionnaire took approximately 45 min to complete. There were no substantial changes suggested by patients to the content of dimensions or items. All items and dimensions were thought to be relevant to the Malawi setting. Suggestions were however made to the local language translation to improve comprehensibility of items in the continuity dimension. A further suggestion concerned timing of interviews to fit better into normal flow of services as patients were waiting to be attended to.

### Study participants

Out of 649 patients approached, 18 (2.8%) declined to participate in the study. These results are based on 631 completed questionnaires. Missing data accounted for approximately 1.9% of all data. Table 2 shows the socio-demographic characteristics of the 631 study participants of which 65.1% were female, 74.1% were under the age of 40 years and 2.7% were above 65 years. Education was generally low with 80.9% having only attended 8 years of primary school or less. We found that 41.7% of the patients were unemployed themselves while 52.5% came from homes where the household head was unemployed. Access to safe water and electricity were major challenges as only 21.9% of households had access to safe water while access to electricity was at 6.3%.

**Table 2:** Sociodemographic characteristics of total study subjects (*N* = 631) and comparison of Sample 1 and 2

	Total sample (*N* = 631)	Sample 1(*n* = 323)	Sample 2 (*n* = 308)	*p* value
Gender				
Male	220 (34.9)	110 (34.4)	110 (35.7)	0.37
Female	411 (65.1)	213 (65.6)	198 (64.3)	
Age (years)				
Up to 40	467 (74.1)	242 (74.9)	225 (73.4)	0.33
41–65	146 (23.2)	75 (23.2)	71 (22.9)	
> 65	18 (2.7)	6 (1.9)	12 (3.7)	
Education				
< 5 years of primary school	271(43.0)	132 (40.9)	139 (45.1)	0.14
6–8 years of primary school	239 (37.9)	128 (39.6)	111 (36.4)	
Attended secondary school	113 (17.9)	60 (18.6)	53 (17.2)	
Post-secondary education	8 (1.3)	3 (0.9)	5 (1.2)	0.36
Employment				
Full time	54 (8.6)	31 (9.6)	23 (7.5)	0.17
Part time	103 (16.3)	52 (16.1)	51 (16.6)	
Self-employed	211 (33.4)	101 (31.3)	110 (35.7)	
Unemployed	263 (41.7)	139 (43.0)	124 (40.2)	0.24
Piped water/protected well nearby within compound or nearby				
Yes	138 (21.9)	69 (21.1)	69 (22.4)	0.35
No	493 (78.1)	254 (78.9)	239 (77.6)	
Electricity in the home				
Yes	41 (6.3)	23 (7.1)	18 (5.8)	0.25
No	590 (93.7)	300 (92.9)	290 (94.2)	
Head of house employment status				
Employed	301 (47.5)	158 (48.9)	143 (46.4)	0.27
Unemployed	330 (52.5)	165 (51.1)	165 (53.6)	
Health status				
Good to Excellent	418 (66.2)	208 (64.4)	210 (68.2)	0.16
Poor to Fair	213 (33.8)	115 (35.6)	98 (31.8)	
Years in contact with HC				
Up to 2 years	154 (24.4)	82 (25.4)	72 (23.4)	0.28
3–4 years	69 (10.9)	30 (9.3)	39 (12.6)	
> 4 years	408 (64.7)	211 (65.3)	197 (64.0)	
Contact times with HC in past 2 years				
0–4 times	215 (34.1)	107 (33.1)	108 (35.1)	0.30
5–9 times	171 (27.1)	81 (25.1)	90 (29.2)	
> 10 times	245 (38.8)	135 (41.8)	110 (35.7)	0.06
Chronic condition				
Yes	254 (39.6)	139 (43.0)	115 (36.7)	0.06
No	377 (60.4)	184 (57.0)	193 (63.3)	

Of the total interviewees, 75.6% had been in contact with their health center for at least 3 years and 65.9% had visited their health center at least 5 times within two years. 39.6% reported having a chronic condition and 33.8% indicated poor to fair health.

Table 2 also shows that the socio-demographic characteristics of sample 1 and 2 had no statistical difference across all parameters.

### Exploratory factor analysis (EFA)

Initially, the factorability of the 106 items was examined on the one half of the data set. Firstly, it was observed that all the items correlated at least 0.3 with at least one other item. Secondly, the Kaiser-Meyer-Olkin measure of sampling adequacy was calculated to be 0.72, above the commonly recommended value of 0.6 and Bartlett’s test of sphericity was significant (χ2 (4278) = 10,951.7, *p* < .01). Finally, the communalities were above 0.3 for 101 items, further confirming that most items shared some common variance with others. Given these overall indicators, factor analysis was deemed to be suitable with all 106 items.

### Construct validity

Results of the rotated matrix after principal axis factoring, varimax rotation and Kaiser normalization are found in Additional file 1. Seven common factors were extracted based on the initial exploratory factor analysis and were named first contact - access, continuity of care (communication), continuity of care (personal relationship), coordination, comprehensiveness (services available), comprehensiveness (services provided) and community orientation. Initial item reduction was based on the scree test and then retaining items with factor loadings of at least 0.32, items sharing the same underlying meaning of construct without cross loadings of the same significance and inter-item correlation between 0.2 and 0.5. As a result, from the preliminary number of items those retained were as follows: 3 of the 18 items in the first contact - access domain, 4 of the 7 items in the continuity of care (communication) domain, 4 of the 9 items in continuity of care (personal relationship) domain, 3 of the 13 items in the coordination domain, 6 of the 28 items in the comprehensiveness (services available) domain, 6 of the 19 items in the comprehensiveness (services provided) domain and 3 items from the community orientation domain.

As shown in Table 3, factor loadings ranged from 0.34 to 0.89. The coordination domains were analyzed separately to include only those patients that had experienced referral.

**Table 3:** Results of exploratory factor analysis^a^ and internal consistency (*n* = 323) of PCAT-Mw

Scale	Number of retained items/original items	Factor loadings on the scale	Item-total correlation range	Cronbach’s alpha
First contact- access	3/18	0.34–0.59	0.31–0.62	0.66
Continuity of care - communication	4/7	0.36–0.62	0.39–0.56	0.73
Continuity of care- personal relationship	4/9	0.47–0.70	0.53–0.63	0.78
Coordination	3/13	0.81–0.89	0.78–0.87	0.91
Comprehensiveness -services available	6/28	0.34–0.52	0.42–0.46	0.71
Comprehensiveness -services provided	6/14	0.50–0.68	0.43–0.59	0.80
Community orientation	3/6	0.41–0. 57	0.49–0.67	0.78
Total	29/95			0.82

^a^Principal axis factoring, varimax rotation

### Internal consistency

The Cronbach’s alpha coefficient results ranged from 0.66 (first contact) to 0.91 (coordination) for all revised multi-item scales. The item-total correlations ranged from 0.31 to 0.87, meeting the acceptable standard of > 0.30 (Table 3).

### Likert scale assumptions

Tables 3 and 4 show the results of Likert scaling assumptions using the seven revised multi-item scales. All item-scale correlations were above the accepted minimum (0.30) with the majority being greater than 0.50. All scales demonstrated a relatively narrow range of item-scale correlations. Five of the seven scales showed 100% discriminant validity. The two comprehensiveness available and comprehensives provided had items that correlated higher in other scales but were retained because of other favorable metric properties.

**Table 4:** Results of item convergent and discriminant validity testing (*n* = 323) of PCAT-Mw

Scale	Number of items	Item- scale correlation	Item- other scale correlation	Scaling success rate (%)
First contact - access	3	0.31–0.65	0.03–0.21	21/21 = 100%
Continuity of care - communication	4	0.46–0.72	0.01–0.41	28/28 = 100%
Continuity of care - personal relationship	4	0.34–0.70	0.10–0.33	28/28 = 100%
Coordination	3	0.69–0.81	0.02–0.41	21/21 = 100%
Comprehensiveness- services available	6	0.33–0.65	0.07–0.39	40/42 = 95%
Comprehensiveness- services provided	6	0.31–0.92	0.03–0.39	46/49 = 94%
Community orientation	3	0.36–0.52	0.05–0.38	21/21 = 100%

### Confirmatory factor analysis (CFA)

**Fig. 2:**
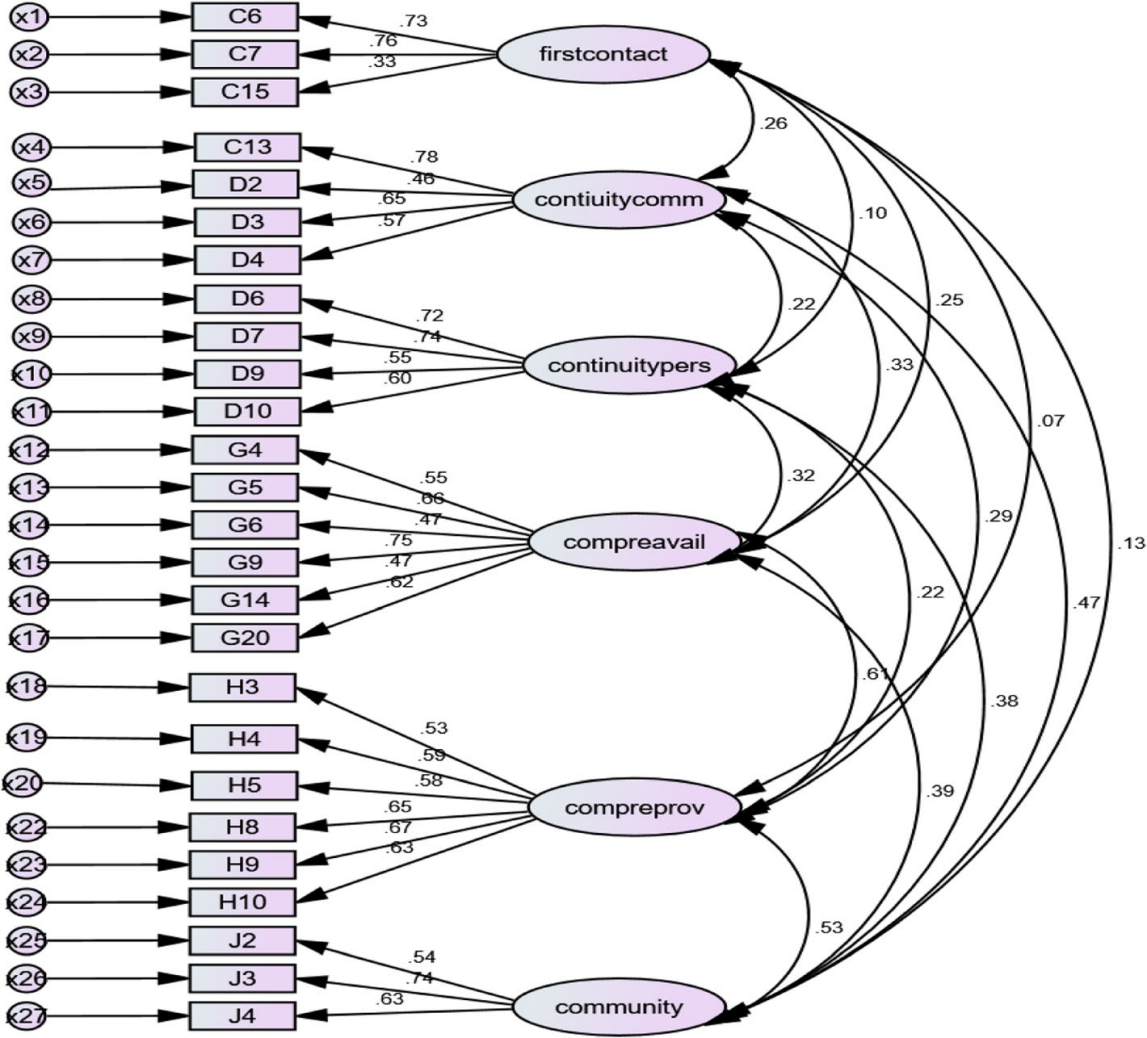
Structural equation model of Sample 2, *n* = 308, with imposed equality constraint of 1 on the factors

The structural equation model (SEM) for sample 2 is illustrated in Fig. 2. After allowing for some covariations between unique variables, this model produced a satisfactory goodness of fit to the model: chi squared test = 462.59, df = 270, CMIN/df = 1.71, *p* = < 0.001, GFI = 0.90, CFI = 0.91, RMSEA = 0.05, PCLOSE = 0.65.

### Descriptive features of PCAT-mw

Table 5 presents estimates of central tendency, dispersion, and other features of the seven revised scales representing four core primary care principles and one derivative domain. The full range of possible scores was observed for all scales. Continuity (personal relationship) and the two comprehensiveness domains were positively skewed, indicating distributions with more negative ratings of primary care. The other four scales were negatively skewed indicating more positive ratings among patients.

**Table 5:** Descriptive features of PCAT-M

Scale	Number of items	Mean	Standard deviation	25th percentile	50th percentile	75th percentile	Range	Skewness	Kurtosis
First contact - access	3	8.48	2.44	7	9	10	3–12	−1.62	0.32
Continuity of care-communication	4	14.53	2.53	9	16	16	4–16	−.2.18	3.84
Continuity of care- personal relationship	4	9.26	4.26	4	7	16	4–16	0.99	−1.68
Coordination	3	9.64	3.43	8	12	12	3–12	−1.14	−0.54
Comprehensiveness – services available	6	14.5	5.01	7	12	23	6–24	0.38	−1.04
Comprehensiveness – services provided	6	22.25	5.86	15	28	28	7–28	1.09	−0.36
Community orientation	3	11.80	3.79	7	16	16	4–16	−0.65	1.36

### Further reliability

Forty four out of 50 patients (88%) returned for a second interview at the intra –rater reliability chosen facility while 42 out of 50 patients (84%) returned for a second interview at the inter – rater chosen facility. A high level of reliability was found between the sum scores of the domain mean scores in both the intra-rater test re-test and the inter-rater reliability. The Intra-class Correlation Coefficient (ICC) for the intra-rater test re-test was 0.90 with a 95% confidence interval (CI) of 0.81–0.95 (*n* = 44, *p* < 0.001). The ICC for inter-rater reliability was 0.84, 95% CI 0.71–0.96 (*n* = 42, *p* < 0.001).

The final version of the adult PCAT-Mw questionnaire is attached as Additional file 2.

## Discussion

This study developed a 29 item PCAT-Mw with seven scales as a tool for measuring the performance of primary care from adult patients’ experience in the Malawian context. The items in the PCAT-Mw measure the four core dimensions of primary care: first contact - access, continuity of care, coordination and comprehensiveness of services as well as the derivative dimension of community orientation. The PCAT-Mw is significantly shorter making it time efficient in administration and will contribute to the evaluation of primary care performance in Malawi.

Accepted methods of cross-cultural adaptation were carried out on the South African version. The resultant PCAT-Mw underwent standard metric analyses to assess reliability and validity. The high ICC observed for both intra-rater and inter-rater reliability could be due to the fact that the PCAT-Mw measures patients’ experience rather than satisfaction with care and that the 4 weeks’ interval was optimal for repeat measurements.

The dimension of coordination was not included in the structural equation model (SEM) because of limited data as only 16% of patients reported to have been referred to a higher level of care. However confirmatory factor analysis performed on the items under first contact - access, continuity of care, comprehensiveness of services and community orientation yielded results that indicated that the retained items sufficiently represented the conceptual multidimensional nature of primary care. Models of these core dimensions and the one derivative dimension of community orientation showed satisfactory statistical fit.

This also supports the idea that the creation of effective primary care systems is context dependent and that the strength of a country’s primary care system is determined by the degree of development of a combination of core primary care dimensions in the context of its health care system [46, 47]. With regards to Likert scale assumptions, the two comprehensiveness scales had some items that correlated with other scales. However, the other five scales achieved 100% item-other scale discriminant validity, and the other Likert scaling assumptions, including item convergent validity, equal item-scale correlation, and score reliability, were satisfied, which suggests by and large the appropriateness of the usage of the Likert scales in this study which can be used without standardization.

PCAT-Mw is different in the factor structure from the original PCAT adult expanded version and ZA-PCAT on which adaptation was based. The original version consists of four core dimensions represented by six scales and three derivative domains while the South African version has an additional derivative domain “the primary care team”. Nonetheless, the final PCAT-Mw scales are consistent with the theoretical four core principles of primary care. While the domain “primary care team” was eliminated at content validity stage, “family orientation” and “cultural competence” did not satisfy metric analysis requirements for retention similar to other studies [30–32].

There are a number of ways in which a reliable and valid tool such as the PCAT-Mw would be applied in health services research. This study shows that although primary care in Malawi is structured differently, it does conform to the accepted definition and reflects the multi-dimensionality as proposed by the Institute of Medicine [35]. The instrument can be used to assess the content and organization of primary care in Malawi in the regions where Chichewa is the main language. Another application is the use of the PCAT-Mw to set the standards of quality of primary care based on data on patients’ experience of service delivery. In this regard, the PCAT-Mw can be used on its own as well as in combination with clinical outcome measures. Users of the PCAT-Mw should review the adequacy and relevance of the comprehensiveness domains to the context in which they are to be applied. Similarly, those items that showed lower item-total correlation may be considered to be used when more information on accessibility is desired.

The study had a number of potential limitations. First is that although an adequate sample size as confirmed by the Kaiser-Meyer-Olkin and Bartlett’s test results, the study was carried out in one rural district, which may limit its generalizability to the national scale particularly in those regions where people largely speak another language other than Chichewa. This currently accounts for about 35% of the population. Cross cultural adaption will be needed when another language should be used. Another potential limitation on generalizability is the exclusion of acutely ill, frail and patients with severe mental illness. Further studies should consider different settings to include patients that initially presented with conditions that needed immediate attention to assess their experience of primary care. Second is the potential for recall bias inherent with this nature of studies. The intra-rater and inter-rater reliability tests and the one to one interviewing sought to ascertain minimal measurement error that would arise from it.

The PCAT-Mw is a new instrument in this setting. However, it is based on a standardized and widely used questionnaire and a full validation procedure was undertaken. Further, future application of the tool in more regions and populations could add to its validation on a wider scale. Future studies could also develop tools for providers, managers and children to provide a comprehensive assessment of primary care as was developed in the original set of tools and could combine this methodology and disease specific quality of care measurement.

## Conclusion

This study indicates that the PCAT Mw is a reliable and valid tool to assess core concepts of primary care as seen from patients’ perspective in Malawi. It can be used to establish baseline and to compare primary care performance from patients’ perspectives over time. Further studies could focus on assessing responsiveness and developing tools for providers, managers and children and to compare measures of patients’ experiences with disease specific outcomes in Malawi.

## Additional files

Additional file 1: Exploratory factor analysis of PCAT-Mw - Rotated factor matrix after principal axis factoring, varimax rotation with Kaiser normalization. This presents the factor loadings of each item and the number of factors extracted after initial factor analysis. (DOCX 35 kb)

Additional file 2: Primary care assessment tool Malawi adult version (PCAT-Mw). This is the final validated PCAT-Mw with 29 items in English and the local language Chichewa and socio-demographic data and health care questions. (DOCX 188 kb)

## Abbreviations

CFA: Confirmatory Factor Analysis
CFI: Comparative Fit Index
CI: Confidence Interval
EFA: Exploratory Factor Analysis
GFI: Goodness of Fit Index
ICC: Intra-class correlation coefficient
PCAT - Mw: Primary Care Assessment Tool – Malawian version
PCAT: Primacy Care Assessment Tool
PCLOSE: *P* value of close fit
RMSEA: Root Mean Squared Error of Approximation
SEM: Structural Equation Model
ZA-PCAT: Primary Care Assessment Tool – South African version
