# Supplementary material for: Development and validation of a Malawian version of the primary care assessment tool
Source: BMC Fam Pract. 2018 May 16;19:63. doi: 10.1186/s12875-018-0763-0 (PMC5956555; doi:10.1186/s12875-018-0763-0)
Supplement: Supplementary file 1 — Exploratory factor analysis of PCAT-Mw - Rotated factor matrix after principal axis factoring, varimax rotation with Kaiser normalization. This presents the factor loadings of each item and the number of factors extracted after initial factor analysis. (DOCX 35 kb) [file 12875_2018_763_MOESM1_ESM.docx]

| Exploratory factor analysis of PCAT-Mw: Rotated factor matrix after principal axis factoring,  varimax rotation with Kaiser normalization ^a^ | | | | | | | | |
| --- | --- | --- | --- | --- | --- | --- | --- | --- |
|  | Factor | | | | | | | |
|  | 1 | 2 | 3 | 4 | 5 | 6 | 7 |  |
| How to prevent falls | .690 | .161 | .083 | .071 | .030 | .168 | .020 |  |
| How to prevent hot burns | .668 | .130 | .075 | .057 | -.016 | .125 | .071 |  |
| ways to handle family conflict; arguments; disagreements (that may arise from time to time) | .592 | .192 | .151 | -.018 | .061 | -.045 | .047 |  |
| Possible exposure to harmful substances in your home, at work or in your area e.g. paraffin; pesticides? | .542 | .188 | .168 | .116 | .051 | -.054 | .124 |  |
| Home safety, like storing medicines safely; safe use of charcoal burner. | .463 | -.057 | -.032 | -.041 | .067 | -.048 | .038 |  |
| Advice about healthy and unhealthy foods | .448 | .033 | -.103 | -.005 | .009 | .096 | -.006 |  |
| Advice about appropriate exercise for you | .438 | .155 | .254 | .005 | .096 | -.040 | .046 |  |
| Checking for weight problems? | .432 | .203 | .246 | .048 | .178 | -.033 | -.051 |  |
| Advice on wearing reflectors when walking on the road at night | .411 | .139 | .241 | .105 | .097 | -.067 | -.051 |  |
| Checking and discussing the medications you are taking | .359 | .108 | .264 | .080 | .195 | -.109 | .158 |  |
| Advice on water, hygiene and sanitation? | .325 | .098 | -.137 | -.054 | -.080 | .136 | .160 |  |
| Allergy injections | .305 | .084 | .225 | .148 | .100 | .145 | -.127 |  |
| Advice and treatment on sexually transmitted infections | .281 | -.087 | .185 | .050 | .019 | -.082 | -.031 |  |
| For females: breast examination. | .226 | -.059 | .217 | .085 | .030 | -.172 | .073 |  |
| Does you HC do surveys of patients to see if services are meeting People’s needs? | .161 | .703 | .074 | -.002 | .088 | .013 | .033 |  |
| Does your HC get opinions and ideas from people or organizations with knowledge to help provide better health care? E.g. the local health committee, churches, other organizations? | .045 | .630 | .068 | .109 | .069 | .036 | .216 |  |
| Does your HC do surveys in the community to find out about health problems it should know about? | .189 | .602 | .030 | .079 | .140 | .132 | .029 |  |
| Do you think your HC knows about the important health problems of your area? | .009 | .521 | .070 | .047 | .128 | .053 | .157 |  |
| What to do in case someone in your family cannot make decisions about his/her care e.g. very old (senile) or severe mental illness. | .379 | .431 | .310 | .132 | .110 | .061 | -.139 |  |
| Does anyone at your HC ever make home visit | .042 | .414 | -.034 | .177 | .005 | .114 | .152 |  |
| Does your HC ask you about your ideas and opinions when planning treatment and care for you or a family member? | .141 | .372 | .245 | .213 | .190 | -.005 | -.118 |  |
| Support when someone in your family has mental or physical disabilities that are normal with getting older e .g. Frail when too old or too disabled by stroke | .312 | .372 | .305 | .053 | .081 | .061 | -.064 |  |
| Counseling to stop smoking | .228 | .336 | .317 | .050 | .188 | .054 | .033 |  |
| Ask members of your community to be on the local Health committee? | .134 | .307 | -.107 | .013 | .097 | .117 | .060 |  |
| Do you feel comfortable discussing religious or Cultural issues that affect your health with staff at the HC? | .150 | .292 | .236 | .277 | .100 | -.201 | .072 |  |
| Checking to see if anyone in your family would benefit from any social support eg OVCs, people living with disability. | .220 | .273 | .139 | .087 | .109 | .082 | -.114 |  |
| Help with food supplements such as Chiponde/Likuni phala or food parcels | .070 | .230 | .043 | .130 | .106 | .140 | -.043 |  |
| Suggestions for home-based care e.g. a visit from a home-based carer? | -.005 | .230 | .042 | .081 | .090 | .054 | .115 |  |
| Dental check-up – checking and cleaning your teeth | .199 | .163 | .478 | .238 | -.003 | .188 | -.090 |  |
| TB Testing | -.004 | .080 | .460 | -.053 | .027 | .139 | .134 |  |
| Plastering fractures | .054 | -.054 | .458 | -.009 | .015 | .205 | -.108 |  |
| Treatment of diabetes mellitus? | .116 | .163 | .447 | -.030 | -.071 | .169 | .419 |  |
| Treatment for an ingrown toenail i.e. removing part of the toenail | .184 | .161 | .433 | .117 | -.035 | .103 | -.006 |  |
| Tests for sugar levels in your blood | .271 | .031 | .424 | .047 | .041 | .068 | .331 |  |
| Checking your hearing | .095 | .309 | .368 | .085 | .150 | .097 | -.091 |  |
| VIA tests for cervical cancer | .075 | .047 | .338 | -.043 | .007 | .051 | .039 |  |
| Counseling for mental health problems | .295 | .285 | .331 | .020 | .131 | .170 | .077 |  |
| Treatment for high blood pressure? | .140 | -.024 | .313 | .003 | .040 | .097 | .289 |  |
| When you come to this HC are you taken care of by the same doctor or clinician or nurse each time? | -.013 | .063 | -.247 | .110 | -.008 | -.049 | -.001 |  |
| Has your HC asked about illnesses or problems that might run in your family? | .061 | .233 | .240 | .158 | .133 | -.108 | .029 |  |
| Does your HC know who lives with you? | .077 | .060 | -.140 | .703 | .009 | .045 | .121 |  |
| Does your HC know you very well as a person, rather than as someone with a medical problem? | .015 | .116 | -.139 | .659 | .021 | .171 | .179 |  |
| Does your HC know your complete medical history? | .007 | .162 | .162 | .654 | .117 | -.096 | .069 |  |
| Does your HC know about your work or employment | .041 | .129 | -.070 | .648 | .027 | -.021 | .006 |  |
| Does your HC know what problems are most important to you? | .052 | .126 | .057 | .556 | .152 | .076 | .079 |  |
| Would your HC know if you had trouble getting the medicines you needed | -.006 | .165 | .205 | .454 | .111 | -.282 | -.061 |  |
| Does your HC know about all the medications you are taking? (e.g. getting elsewhere including traditional medicines) | .095 | -.050 | .159 | .359 | .142 | -.200 | -.087 |  |
| When your HC is closed is there a phone number you can call when you get sick? | .053 | .053 | .209 | .216 | .205 | .073 | -.084 |  |
| Do you think the staff at this HC understands what you say or ask? | .074 | .022 | -.008 | .015 | .645 | .066 | .019 |  |
| Are your questions answered in a way that you understand? | .232 | .150 | .052 | .021 | .628 | .037 | .029 |  |
| Does your HC give you enough time to talk about your problems or worries | .091 | .113 | -.008 | .151 | .587 | .095 | -.027 |  |
| Is the staff friendly and approachable? | .025 | .206 | .024 | .029 | .526 | .088 | .009 |  |
| Do you think your HC understands/respects your culture? | .042 | .105 | .130 | .062 | .488 | -.111 | .178 |  |
| Is it easy to lay a complaint or compliment or make a suggestion at your HC | .025 | .110 | .082 | .131 | .419 | .182 | .135 |  |
| Are you comfortable talking to a doctor or clinician nurse at your HC | .016 | .065 | -.007 | .074 | .368 | -.002 | .066 |  |
| Do you always get the services you need the same day or do you have to come back another day because the services are not offered on the same day? | -.003 | .092 | .107 | -.047 | .233 | .111 | .189 |  |
| When your HC is closed and you get sick during the night, would someone from there see you that night? | -.119 | .001 | .186 | -.049 | .241 | .610 | -.099 |  |
| When your HC is closed on Saturday and Sunday and you gwt sick, would someone from there see you the same day? | .061 | -.066 | .172 | .060 | .205 | .604 | -.074 |  |
| Incision and drainage of abscess | .011 | .054 | .139 | .038 | -.021 | .513 | .083 |  |
| Stitching up a cut that needs stitches | .196 | .094 | .093 | .010 | -.019 | .438 | .102 |  |
| Is your HC open in the evenings for at least some weekdays? | -.070 | .042 | .025 | -.155 | .015 | .407 | -.065 |  |
| When your HC is open and you get sick, would you be seen the same day? | -.060 | .057 | .060 | -.003 | .138 | .372 | .104 |  |
| Is there a complaints / suggestion box at your HC? | .079 | .079 | .208 | .069 | .085 | .333 | .023 |  |
| Ante-natal care i.e. care for pregnant mothers | .107 | .138 | -.010 | -.006 | -.124 | .283 | .080 |  |
| If it was easy to do, would you change your HC to somewhere else? | .050 | .180 | -.150 | .150 | .175 | .275 | .244 |  |
| Treatment of malnutrition? | .087 | .178 | .047 | .040 | -.006 | .265 | -.014 |  |
| For females: care for common menstrual or menopause problems | .048 | -.069 | .054 | .052 | .047 | -.127 | .007 |  |
| Would you recommend your HC to a friend or relative? | -.063 | .040 | .018 | .044 | .156 | -.004 | .520 |  |
| Would you recommend this HC to someone who does not speak your home language well? | .036 | .077 | .052 | .121 | -.005 | -.014 | .515 |  |
| Would you recommend your HC to someone who uses traditional medicine or home remedies such as garani, moringa herbs, or has special beliefs about health care? | .096 | .132 | .016 | .005 | .155 | -.007 | .447 |  |
| Can you change your HC if you wanted to? | .126 | .129 | -.091 | .135 | .152 | .169 | .225 |  |
| Treatment for malaria | -.009 | -.030 | -.045 | .037 | -.080 | .073 | .125 |  |
| Counseling and testing for HIV/AIDS | .135 | -.049 | -.003 | -.070 | .131 | .035 | -.061 |  |
| Is it easy to be seen for a general checkup at your HC | .040 | .027 | -.064 | .052 | .176 | .042 | .095 |  |
| Would your HC meet with members of your family if you thought it would be helpful? | .133 | .008 | .008 | .064 | .091 | -.077 | .114 |  |
| a. Rotation converged in 11 iterations. | | | | | | | | |
