# Supplementary material for: Development and validation of a Malawian version of the primary care assessment tool
Source: BMC Fam Pract. 2018 May 16;19:63. doi: 10.1186/s12875-018-0763-0 (PMC5956555; doi:10.1186/s12875-018-0763-0)
Supplement: Supplementary file 2 — Primary care assessment tool Malawi adult version (PCAT-Mw). This is the final validated PCAT-Mw with 29 items in English and the local language Chichewa and socio-demographic data and health care questions. (DOCX 188 kb) [file 12875_2018_763_MOESM2_ESM.docx]

**PRIMARY CARE ASSESSMENT TOOL MALAWI ADULT VERSION (PCAT-Mw)**

Originally developed by **Barbara Starfield, MD, MPH** Primary Care Policy Center

School of Hygiene and Public Health Johns Hopkins University Baltimore, USA

Adapted and validated for use in Malawi by the PCAT Study Research Team, Partners In Health 2016 as

**PCAT-Mw**

ENGLISH AND CHICHEWA VERSION

**ADMINISTRATIVE INFORMATION**

Interviewers name: __-----------------------------------------------------------------------------

Date……………………………… Time interview began ……………….. ……..Time interview finished ………………………………

**INTRODUCTION/SCREENING QUESTIONS**

__________________________

*RECRUITING & PURPOSE OF THE SURVEY.*

*Interviewer: Hello, my name is I’m working with other colleagues from …………….conducting a survey asking patients what they think about the health care they receive. All the information given is private and confidential and will remain anonymous. We are not recording your name and address on the survey form. We only require your name and signature on the consent form to show the …………(IRB) that we have asked for your permission and you have agreed to be part of the study.*

*Would you be willing to answer a few questions about your experience of health care while you are waiting?*

*1 Yes If Yes, in which language would you prefer to speak? (Go to separate consent form)*

*2 No If No, terminate interview by saying: Thank you for your time. I apologize for any inconvenience.*

**CHOLINGA CHA KAFUKUFUKUYI.**

**Ofunsa mafunso: Muli bwanji? dzina langa ndine……..Ndikugwira ntchito ndi …………… omwe akupangitsa kafukufuku kwa odwala za maganizo awo pa chithandizo chomwe amalandira pachipatala chakudera lawo. Ndipo tikukutsimikizirani kuti chilichonse chomwe muyankhule ndi cha chinsinsi ndipo dzina lanu silidzatchulidwa mu njira ina iliyonse. Kotero kuti sitilemba dzina lanu kapena kumene m’machokera mu fomu yakafukufukuyi. Dzina lanu ndi kusayinira zipezeka pa fomu yokhayo yosonyeza ku ………..(IRB) kuti tapempha chilolezo chanu ndipo mwavomereza kutengapo gawo pakafukufuku ameneyi.**

**Kodi mulola kuyankha mafunso ochepa okhudzana ndi mbiri yanu ndi m’mene m’mathandizidwira pachipatala chino pamene mukudikira kuthandizidwa?**

**1 ….Eya. Ngati avomera afunseni chiyankhulo chomwe angamasuke nacho kuyankhula**

**2. … No. Ngati sanasangalatsidwe, musapitilize kuwafunsa mafunso. Athokozeni chifukwa chanthawi yopambana komanso muwapepese ngati izi zawasokoneza mu njira ina iliyonse.**

*AFTER CONSENT COMPLETED:*

*THANK YOU FOR AGREEING TO ANSWER A FEW QUESTIONS ON YOUR EXPERIENCE OF HEALTH CARE.*

*FIRST, I WOULD LIKE TO ASK YOU A FEW GENERAL QUESTIONS BEFORE ASKING ABOUT YOUR EXPERIENCE.*

**MUKAMALIZA MAFUNSO OFUNSIDWA PACHIYAMBI OFUNA KUDZIWA NGATI ALI OMASUKA KUTENGA NAWO GAWO PAKAFUKUFUKUYI:**

**ZIKOMO KWAMBIRI POVOMERA KUTENGAPO GAWO POYANKHA MAFUNSO OCHEPA PA MBIRI YANU YA PACHIPATALA CHINO. CHOYAMBIRIRA NDIKUFUNSANI MAFUNSO ENA NDI ENA NDISANAKUFUNSENI ZA MBIRI YANU NDI CHITHANDIZO CHOMWE M’MALANDIRA PA CHIPATALA PANO.**

*A. EXTENT OF YOUR AFFILIATION (RELATIONSHIP) WITH A PRIMARY CARE PLACE OR PERSON*

*(HC/ CLINIC / HOSPITAL / GENERAL PRACTICE / DOCTOR / NURSE)*

MULINGO WA UBALE WANU NDI MALO A CHITHANDIZO KAPENA MUNTHU (PACHIPATALA PANO/ZOCHITIKA PANO/ NDI A DOTOLO KAPENA A NAMWINO

*A1. Where do you usually go when you are ill or need to talk to someone about your health? Please give the name of*

*the place or person:/* **Ndikuti kumene m’makonda kupita mukadwala kapena mukafuna kukamba zokhudza umoyo wanu? Chonde tchulani dzina la malowo kapena munthuyo:**

A2. *Is there another place / person you sometimes go for health care?*/ **A2. Kodi palinso malo ena kapena munthu winanso kumene nthawi zina m’mapita?**

a  No/**AYI**

*b  Yes…………………………………………. Please give name of place or person:*

**Eya…………………………………………..chonde tchulani dzina la munthuyo kapena malowo**

A3. *Which place / person knows you best regarding your health care?* ***Ring*** *A1 or*  **A2/ Ndi ndani kapena ndi malo ati omwe amadziwa bwino lomwe za umoyo wanu? Onani A1 kapena A2**

*For the interviewer : ‘YOU HAVE BEEN TO THIS HC 3 TIMES OR MORE. ALL THE QUESTIONS ARE ABOUT YOUR EXPERIENCE OF PRIMARY CARE AT THIS HC.’*

**Chigawo cha ofunsa mafunso:**

**Mwafikapo pachipatalachi katatu konse kapena kupitiriranso. Mafunso otsatirawa akukamba za mbiri ya zomwe mwakumana nazo nokha za kathandizidwe ka odwala pachipatalachi**

A4  *Which of these can be seen at thisHC?*

*1 Adults only 2 Both children and adults 3 Only certain kinds of problems 4 Most kinds of problems*

*9 Not sure/don’t remember*

**Ndi anthu odwala matenda anji omwe amapezeka pachipatalachi?**

**1…..Akuluakulu okha 2…...Ana ndi akuluakulu 3….. a mavuto owerengeka 4….a mavuto ambiri**

**5……sindikudziwa/sindikukumbukira**

*A5 About how many times in the last 2 years have you been to this HC? Times*

**Kodi mwabwerako kangati pachipatalachi mu zaka ziwiri zapitazi? kangati_________________________________**

A6 *How long have you been coming to this HC?* **kodi mwakhala mukubwera kuyambira liti**

*1 Less than 6 months*

*2 Between 6 months and one year*

*3 1 - 2 years*

*4 3 - 4 years*

*5 5 or more years*

*6 Difficult to say (too variable to specify)*

*7 Not sure/don’t remember*

**Mwakhala mukubwera pachipatalachi kwa nthawi yayitali bwanji?**

**1 Kosapitilira miyezi isanu ndi umodzi**

**2 Pakati pa miyezi isanu ndi umodzi ndi chaka chimodzi**

**3 Pakati pachaka chimodzi ndi ziwiri**

**4 Pakati pa zaka zitatu ndi zinayi**

**5 Pakati pazaka zisanu kapena kuposela apo**

**6 Ndizovuta kutchula**

**7 sindikudziwa/sindikukumbukira**

**A7** *Did you choose this HC yourself?*

*1 Yes. 2 No 3 Other 9 Not sure/don’t remember*

**Kodi munasankha chipatalachi nokha?**

**1 Eya 2 Ayi 3 Zina 9 sindikudziwa/ sindikukumbukira**

*A8 Do you come to this HC mainly because of a special medical problem? E.g. Hypertension; Diabetes etc*

*1 Yes 2 No 3 Other 9 Not sure/don’t remember*

**Kodi mumabwera pachipatalachi chifukwa cha nthenda yapadeladela? Mwachitsanzo kuthamanga (BP); nthenda**

**ya shuga, mtima, HIV, ndi zina zotero**

**1 Eya 2 Ayi 3 Zina 9 sindikudziwa/ sindikukumbukira**

**B. FIRST CONTACT – ACCESS**

| **Please choose the ONE best answer** | | **Defintely** | **Probably** | **Probably**  **not** | **Definately**  **Not** | **Not sure** |
| --- | --- | --- | --- | --- | --- | --- |
| **B1** | *When this HC is closed on Saturday and Sunday and*  *you get sick, would someone from here see you the*  *same day?*  **Nanga chikakhala chotseka satade ndi sande(loweluka/lamulungu) ndipo inu mwadwala, munthu ochokera kuchipatalachi amakhoza kukuthandizani tsiku lomwelo?** | 4 | 3 | 2 | 1 | 9 |
| **B2** | *When the HC is* ***closed*** *and you get sick* ***during the night,*** *would someone from here see you that night?*  **Chipatala chino chikakhala chotseka ndipo inu mwadwala nthawi ya usiku, munthu ochokera kuchipatalachi amakhoza kukuthandizani usiku omwewo?** | 4 | 3 | 2 | 1 | 9 |
| **B3** | *Is there a complaints / suggestion box at this HC?*  **Kodi pali bokosi loponyamo madandaulo kapena ndamanga zanu pamalo pano?** | 4 | 3 | 2 | 1 | 9 |
|  |  |  |  |  |  |  |

**C. ONGOING CARE**

**Please choose the ONE best answer/**

|  | | | **Defintely** | **Probably** | | **Probably**  **not** | | **Definately**  **Not** | **Not sure** | |
| --- | --- | --- | --- | --- | --- | --- | --- | --- | --- | --- |
| **C1** | | *Is the staff friendly and approachable?*  **Kodi ogwira ntchito pachipatala pano ndi omasuka komanso ofikilika?** | 4 | 3 | 2 | | 1 | | 9 |  |
|  | | |  |  | |  | |  |  | |
| **C2** | *Do you think the staff at this HC understands what you say or ask?*  **Kodi mumaona ngati ogwira ntchito pa chipatala pano amamvetsa zomwe mukunena kapena mukufunsa?** | | 4 | 3 | | 2 | | 1 | 9 | |
| **C3** | *Are your questions answered in a way that you understand?*  **Nanga mafunso anu amayankhidwa munjira imene inuyo mumamvetsetsa?** | | 4 | 3 | | 2 | | 1 | 9 | |
| **C4** | *Does this HC give you enough time to talk about your problems or worries*  **Kodi mumapatsidwa nthawi yokwanira kuti mufotokoze nkhawa ndi mavuto anu?** | | 4 | 3 | | 2 | | 1 | 9 | |
| **C5** | *Does this HC know you very well as a person, rather than as someone with a medical problem?*  **Kodi apachipatalachi amakudziwani bwinobwino ngati munthu osangati ngati munthu odwala?** | | 4 | 3 | | 2 | | 1 | 9 | |
| **C6** | *Does this HC know who lives with you?*  **Nanga amadziwa amene mumakhala nawo** | | 4 | 3 | | 2 | | 1 | 9 | |
| **C7** | *Does this HC know your complete medical history?*  **Kodi chipatalachi chimadziwa mbili yonse ya umoyo wanu?** | | 4 | 3 | | 2 | | 1 | 9 | |
| **C8** | *Does this HC know about your work or employment*  **Nanga ntchito yomwe mumagwira, ogwira ntchito pa chipatalachi amaidziwa?** | | 4 | 3 | | 2 | | 1 | 9 | |

**D. CO –ORDINATION**

**Please choose the ONE best answer**

|  | | | **Defintely** | | **Probably** | | **Probably**  **Not** | | **Definately**  **Not** | | **Not sure** |
| --- | --- | --- | --- | --- | --- | --- | --- | --- | --- | --- | --- |
| **D1** | *Does this HC know what the results of them*  *visit were?*  **Nanga chipatala chino chikudziwa zotsatira za ulendowo?** | 4 | | 3 | | 2 | | 1 | | 9 | |
| **D2** | *After you went to the specialist or hospital did this HC talk with you about what happened at that visit?*  **chipatala chino chinakufunsani za momwe munayendera Mutachoka kokumana ndi dotolo wamkuluyo kapena ku chipatala chachikulucho?** | 4 | | 3 | | 2 | | 1 | | 9 | |
| **D3** | *Does this HC seem interested in the quality of*  *care you get from that specialist or hospital?*  **Nanga chipatala chino chikuonetsa chidwi pa mtundu wachithandizo chomwe mumalandila kuchokera kwa dotolo kapena chipatala chachikulucho?** | 4 | | 3 | | 2 | | 1 | | 9 | |

**E. COMPREHENSIVENESS (SERVICES AVAILABLE)**

**Please check the ONE best answer**

|  | | **Defintely** | **Probably** | **Probably**  **Not** | **Definately**  **Not** | **Not sure** |  |
| --- | --- | --- | --- | --- | --- | --- | --- |
| *Following is a list of services that you or your family might need*  *At some time. For each one, please indicate whether it is available*  *At this HC*  **Zotsatirazi ndi zithandizo zomwe inu kapena aliyense m’banja mwanu angazisowe nthawi ina/iliyonse. Onetsani ngati izi zimapezeka pa chipatala chanu kapena ai** | | |  |  |  |  |  |
| **E1** | | *Checking your hearing*  **Kupima mamvedwe anu** | 4 | 3 | 2 | 1 | 9 |
| **E2** | | *Dental check-up – checking and cleaning your*  *Teeth*  **Kupima ndi kutsuka mano** | 4 | 3 | 2 | 1 | 9 |
| **E3** | | *Treatment by a dental therapist e.g. extractions of bad teeth or dental fillings***.**  **Kuthandizidwa ndi odziwa za mano (monga kuchotsa ndi ku mata mano obooka.)** | 4 | 3 | 2 | 1 | 9 |
| **E4** | | *Counseling for mental health problems*  **Uphungu okhudza matenda ogwira ubongo (monga misala)** | 4 | 3 | 2 | 1 | 9 |
| **E5** | | *Plastering fractures*  **Kuikidwa zikhakhapa mafupa otchoka** | 4 | 3 | 2 | 1 | 9 |
| **E6** | | *Treatment for an ingrown toenail i.e. removing*  *part of the toenail*  **kuchotsa zikhadabo zakumwendo zosamera bwino** | 4 | 3 | 2 | 1 | 9 |
|  |  |  |  |  |  |  |  |

**F. COMPREHENSIVENESS (SERVICES PROVIDED)**

**The next questions deal with different types of health care services that you sometimes get.**

**Please check the ONE best answer**

| **Mafuso otsatilawa ndiokhudza zina zimene mutha kulandira nthawi zina** | | **Defintely** | **Probably** | **Probably**  **not** | **Definately**  **Not** | **Not sure** |  |
| --- | --- | --- | --- | --- | --- | --- | --- |
| *In your visits to this HC, are any of the following*  *subjects discussed with you?*  **Mukamabwera kuchipatala, kodi nkhani izi zimakambidwa?** | | | | | | | |
| **F1** | | *Advice on wearing reflectors when walking on the road at night*  **Malangizo ovala zovala zowala poyenda usiku** | 4 | 3 | 2 | 1 | 9 |
| **F2** | | *Ways to handle family conflict; arguments; disagreements (that may arise from time to time)*  **Njira zothetsera kuyambana m’banja monga kutsutsana, kusamvetsetsana komwe kumachitika nthawi ndi nthawi** | 4 | 3 | 2 | 1 | 9 |
| **F3** | | *Advice about appropriate exercise for you*  **Malangizo okhudza masewero olimbitsa thupi oyenera kwa inu** | 4 | 3 | 2 | 1 | 9 |
| **F4** | | *Possible exposure to harmful substances in your home, at work or in your area e.g. paraffin; pesticides?*  **Kuthekera kopezeka pa malo pamene pamakhala mankhwala oopsa kunyumba ngakhalenso kumalo anu a ntchito (monga mafuta a nyali kapena mankhwala ophera tizilombo)** | 4 | 3 | 2 | 1 | 9 |
| **F5** | | *How to prevent hot burns*  **Kapewedwe ka ngozi ya moto.** | 4 | 3 | 2 | 1 | 9 |
| **F6** | | *How to prevent falls*  **Kupewedwe ka ngozi zakugwa** | 4 | 3 | 2 | 1 | 9 |

|  |  |  |  |  |  |
| --- | --- | --- | --- | --- | --- |

**G.. COMMUNITY ORIENTATION**

**Please check the ONE best answer**

|  | | **Defintely** | **Probably** | **Probably**  **not** | **Definately**  **Not** | **Not sure** |  |
| --- | --- | --- | --- | --- | --- | --- | --- |
| **G1** | | *Do you think this HC knows about the important*  *health problems of your area?*  **Kodi mumaona ngati chipatala chino chimadziwa za mavuto a zaumoyo akuluakulu amene ali mu dera lanu?** | 4 | 3 | 2 | 1 | 9 |
| **G2** | | *Does this HC get opinions and ideas from people or organizations with knowledge to help provide better*  *health care? E.g. the local health committee, churches, other organizations?*  ***Kodi chipatala chino chimatenga maganizo a anthu kapenanso mabungwe odziwa kuti athandize pakupeleka chithandizo cha umoyo chabwino? (monga ma tchalitchi, komiti ya za umoyo)*** | 4 | 3 | 2 | 1 | 9 |
| **G3** | | *Does this HC do surveys of patients to see if services are meeting people’s needs?*  **Kodi chipatalachi chimapanga kafukufuku oona ngati odwala akulandira chithandizo choyenera ndi mavuto awo?** | 4 | 3 | 2 | 1 | 9 |

Please check the  **one** best answer

**H. HEALTH ASSESSMENT**

*H1 Would you say your health is:*

**Kodi umoyo wanu wa tsiku ndi tsiku mungaufotokoze bwanji?**

1 Excellent 2 Very good 3 Good 4 Fair 5 Poor

**Lilibwinno kwambiri Lilibwino Lilibwinoko Lilibwino pang’ono Sililibwino**

H2 *Do you have any physical, mental, or emotional problem that has lasted or is likely to last longer than one year?*

**Kodi muli ndivuto lina lili lonse la m’thupi, la ubongo kapena vuto lomwe mwakhala nalo kwa chaka kapena lomwe mukuona ngati**

litha chaka?

1 Yes 2 No 9 Not sure/don’t remember

Eya Ayi Sindikudziwa, ndaiwala

**I. DEMOGRAPHIC & SOCIOECONOMIC CHARACTERISTICS**

*These are several questions about you and your family***. Tsopano ndikufusani mafuso okhuza za inu ndi banja lanu,**

I1 1 Male 2Female (Tick)

*I2 How old are you? Years /***Muli ndi zaka zingati** *……….*

I3  *In what village, Traditional Authority and district do you live*?

**Mumachokera boma liti, mfumu yailkulu yanu ndindani komanso mudzi uti**

**Boma**…………………….…………..T/a………….……………………VG………………………………………

I4 *In what country were you born*?/ **muna bwadwira dziko liti**

…………………………………………….

I5 *What is your home language*?/**chilankhulo cha kwanu ndi chani**

1. Chichewa 

2. Chisena 

3. Chiyawo 

4. Other

98 Refuse to Answer 

I6 *Do you speak any other language*(s)?/**Mumayankhula zinenero zina**?

1. Yes 

2. No 

98 Refuse to Answer 

I7 *What is this language?*/ **Monga chinenelo chiti**

I8 *Which of the following best describes your work situation now?* **(Choose one) kodi mumagwira ntchito yanji?**

1. *Employed full-time*  **Olembedwa ntchito**

2. *Employed part-time* **ogwira ntchito mwa ganyu**

3. *Self-employed (informal sector*)  **Ntchito yodzilemba ndekha**

4. *Self-employed (formal sector)* 

5. *Student*  **Mwana wa sukulu**

6. *Homemaker*  **okonza pakhomo**

7. *Retired / pensioner*  opuma pantchito

8. *Disabled*  wachilema

9. *Unemployed*  Osagwira ntchito

98 Refuse to Answer 

I9 What is the highest grade that you completed at school? **kodi sukulu yanu munapita nayo patali bwanji**

0 *Did not attend school*  **sindinapite ku sukulu**

1. Std 5 or less  **kulekeza sitandade 5 kutsika mmusi**

2.  *Std 6 to 8 (completed primary school with/without certificate)* **ndinamaliza pulaimale koma satifiketi ndilibe**

3. *Secondary school with/without school certificate of secondary education*  **ndinamaliza secondary**

4. *Completed technical training*  **ndinaphunzira ntchito ya manja**

5. *Have some college/university education, without completing a degree/diploma* **ndinapitako ku koleji koma**

**sindinatenge digili kapena dipuloma**

6. *Completed a degree or diploma*  **ndinatenga digili/dipuloma**

98 Refuse to Answer 

I10  *Do you have piped water in your house?* /**muli ndi madzi a pa mpopi kunyumba kwanu?**

1 Yes **If yes, go to N13**

2 No

98 Refuse to Answer 

I11  *Do you have piped or protected well water in your yard*? **Muli ndi chitsime chowaka kunyumba kwanu**?

1. Yes **If yes, go to N13**

2. No

98 Refuse to Answer 

I12 *Do you have piped or protected well water nearby*? **Muli ndi chitsime chowaka pafupi**?

1. Yes

2. No

98 Refuse to Answer 

I13 *Do you have electricity in your home?*/ *muli ndi magetsi mnyumba mwanu?*

1. Yes

2. No

98 Refuse to Answer 

I14 *Which of the following best describes your dwelling?* **(Choose one) kodi ndi ziti mwa izi**

**zomwe zikufotokoza nyumba yanu mmene ilili?**

1 *Traditional dwelling with grass thatch* /**nyumba yofolera ndi udzu**

2 *Brick house or house with iron sheets* /**nyumba yanjerwa yofolera ndi malata**

3 Other

98 Refuse to Answer 

I15 *Is the head of your household employed?* **Kodi munthu amene amakuyanganirani kapena mutu**

**wapabanjapo amagwira ntchito?**

1.  **Yes**

**2. No**

**98 Refuse to Answer **

**THANK YOU VERY MUCH FOR ANSWERING THESE QUESTIONS TO HELP IMPROVE HEALTH SERVICES**
